# Duloxetine-Induced Liver Injury: A Case Report

**DOI:** 10.7759/cureus.13715

**Published:** 2021-03-05

**Authors:** Bilal Malik, Basel Abdelazeem, Taylor Revere, Nischit Baral, Arvind Kunadi

**Affiliations:** 1 Internal Medicine, McLaren Health Care, Michigan State University, Flint, USA; 2 Medical Education, Michigan State University College of Human Medicine, Flint, USA; 3 Internal Medicine/Nephrology, McLaren Health Care, Michigan State University, Flint, USA

**Keywords:** transaminitis, duloxetine, antidepressant, hepatitis, liver injury, lfts, inflammation

## Abstract

We present a case of a young (43-year-old), otherwise healthy, female patient who developed drug-induced liver injury (DILI) secondary to routine use of duloxetine. Our case aims to raise awareness amongst physicians about the possibility of duloxetine-induced liver injury. We recommend monitoring liver function tests (LFTs) when initiating or adjusting the dose of duloxetine, and intermittently thereafter, to facilitate early identification and management of potential DILI.

## Introduction

Drug-induced liver injury (DILI) is a rare entity with an incidence rate of 19 per 100,000 treated individuals [1]. DILI and acetaminophen overdose are the most common causes of acute liver failure in the United States, 13% and 39%, respectively, compared to 12% for viral hepatitis A and B combined [2]. The drugs typically implicated include, but are not limited to, pain medications, various anti-microbials, and central nervous system agents [3,4].

Duloxetine, a selective serotonin and norepinephrine reuptake inhibitor (SSNRI), is approved to treat depression, anxiety, and neuropathic pain, but is rarely reported to cause hepatic injury in healthy individuals. We present a rare case of duloxetine-induced hepatotoxicity in an otherwise healthy adult female. Our case aims to raise awareness amongst physicians about the possibility of duloxetine-induced liver injury. We recommend monitoring liver function tests (LFTs) when initiating or adjusting the dose of duloxetine, and intermittently thereafter.

## Case presentation

A previously healthy 43-year-old Caucasian female with a past medical history of fibromyalgia, narcolepsy, and chronic pain syndrome presented to the emergency department with gradually worsening pain in the epigastric and right hypochondriac regions over a six-day period. She described the pain as mild to moderate, exacerbated by eating but not coughing, breathing, defecation, urination, or menses. She denied any prior history of gastrointestinal (GI) complaints, family history of a hepatobiliary disease or liver transplant, and alcohol or drug use. She reported associated malaise, decreased appetite, and moderate nausea. She had several episodes of vomiting, constipation, and non-painful, dark-colored urine. The patient’s home medications included duloxetine 90 mg once daily, modafinil 200 mg twice daily, and multiple vitamins. She had been on the same dose of duloxetine for five years prior to the onset of this episode and denied having any recent changes made to her medication regimen. She denied alcohol, non-steroidal anti-inflammatory drugs, acetaminophen, aspirin, and recent antibiotic use. Additionally, she denied any exposure to sexually transmitted infections and contaminated food or water. 

On physical examination, her vitals were stable, though she appeared to be in moderate distress. Her skin was jaundiced, and scleral icterus was evident. Lung and cardiac examinations were clear to auscultation bilaterally, with a regular rate and rhythm, without murmurs, gallops, or rubs, respectively. On abdominal examination, she was mildly tender to palpation in the epigastric and right hypochondriac regions, with no guarding, rebound tenderness and a negative Murphy’s sign. On neurological examination, she was alert and oriented with no notable asterixis in outstretched arms.

Routine labs were performed, including complete blood count (CBC) and complete metabolic profile (CMP), and were all within normal limits except for her hepatic enzymes. Alanine transaminase (ALT), aspartate transaminase (AST), alkaline phosphatase, ammonia, and total bilirubin were elevated, as displayed in Table 1. Additionally, lipase, prothrombin, and international normalized ratio (PT/INR) were within normal limits, and her blood alcohol was zero. Urinalysis was abnormal, with orange-colored urine, moderate bilirubin, and trace albumin, but negative white blood cells (WBC), red blood cells (RBC), and nitrates. Urine pH was in the normal range.

**Table 1:** Laboratory work up results at admission and at the day of discharge CBC: complete blood count; WBC: white blood cells; CMP: complete metabolic profile; AST: aspartate transaminase; ALT: alanine transaminase; INR: international normalized ratio; IgG: immunoglobulin G; IgM: immunoglobulin M.

	Initial lab values (Hospital day 1)	Discharge lab values (Hospital day 11)	Normal Range
CBC			
WBC Count	7.03	7.81	4.5-10x10*3/uL
CMP			
AST	1347	770	13-35 U/L
ALT	3365	1361	13-35 U/L
Alkaline Phosphatase	237	240	41-126 U/L
Total Bilirubin	7.3	5.4	0.2 to 1.2 mg/dL
Ammonia Venous	38	39	11032 umol/L
Iron	251	-	50-179 ug/dL
Total Iron Binding Capacity (TIBC)	373	-	228-460 ug/dL
Iron Saturation Percent	67.29	-	12.00-45.00 % saturation
Ferritin	2542.7	2190.5	10.0-291.0 ng/mL
Prothrombin Time	10.4	10.2	9.5-12.0 sec
INR	1.00	0.94	0.88-1.11
Lipase	194	-	79-393 U/L
OTHER			
Alpha-1 Antitrypsin	179	-	99.0-242.0 mg/dL
Alpha-fetoprotein Tumor Marker	3.4	-	0.0-7.9 ng/mL
Anti-Mitochondrial Antibody	4.6	-	<=20 Units
Anti-nuclear Antibody	Negative	-	Negative
Ceruloplasmin	31.3	-	20.0-60.0 mg/dL
Hemochromatosis, Hereditary DNA	Negative for C282Y and H63D variants in HFE gene	-	Negative
Smooth Muscle (F-actin) IgG Antibody	9	-	<20 Units
Covid-19 Antigen	Negative	-	Negative
Hepatitis A IgM	Non-reactive	-	Non-reactive
Hepatitis B Surface Antigen	Non-reactive	-	Non-reactive
Hepatitis B Core Antibody IgM	Non-reactive	-	Non-reactive
Hepatitis C Antibody	Non-reactive	-	Non-reactive

An ultrasound of the gall bladder was significant for a contracted gallbladder. No gallstones were present. CT of the abdomen and pelvis was unremarkable, with no inflammatory process identified.

The patient was admitted to the hospital for further evaluation and treatment of acute liver failure. She received IV fluids, lactulose for mildly elevated ammonia levels, and her home medications were held. During her admission, gastroenterology was consulted for further evaluation of her abdominal complaints. An abdominal X-ray was performed and negative for obstruction. She received a suppository, which effectively improved her constipation. Esophagogastroduodenoscopy was performed for progressive reflux symptoms. She was started on a trial of proton pump inhibitor (PPI) therapy with relief of her symptoms. Additionally, infectious disease was consulted for an episode of fever as high as 100.8 F. Due to an absence of infectious etiology, a normal WBC count, negative severe acute respiratory syndrome coronavirus 2 (SARS-CoV-2) testing, and a negative hepatitis panel, the fever was concluded to be secondary to inflammatory changes; as such, no antimicrobial therapy was initiated. Her fever subsequently resolved in 24 hours without intervention.

**Figure 1:**
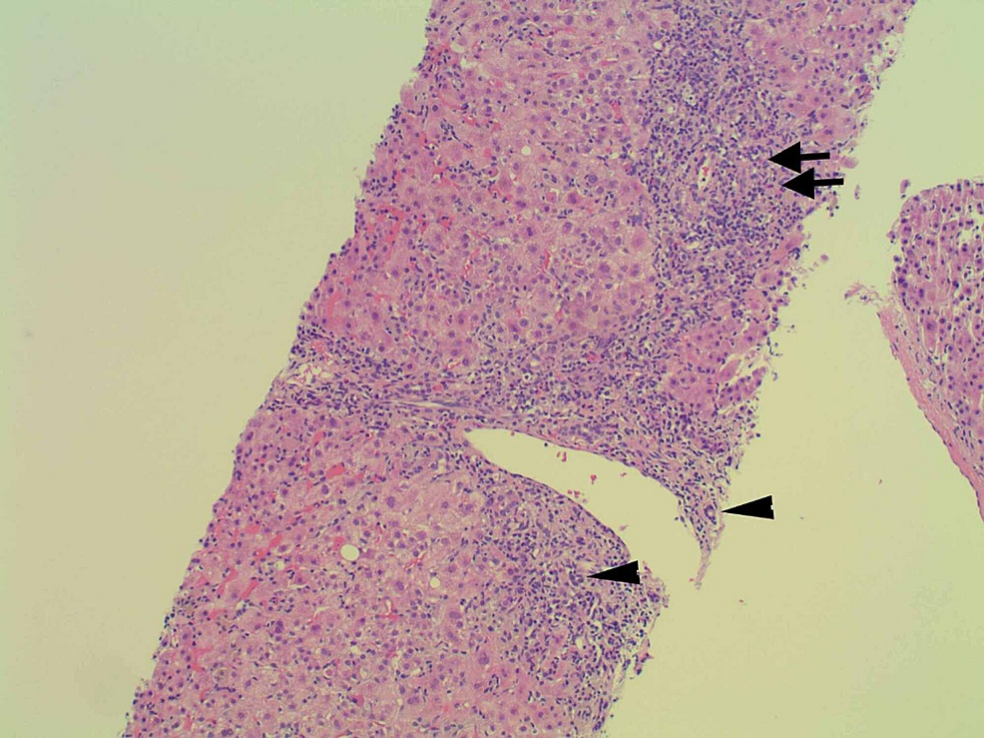
Liver biopsy slide under medium power magnification demonstrating portal, periportal, and patchy lobular hepatitis Arrows demonstrate eosinophils. Arrow heads identify bile ducts.

**Figure 2:**
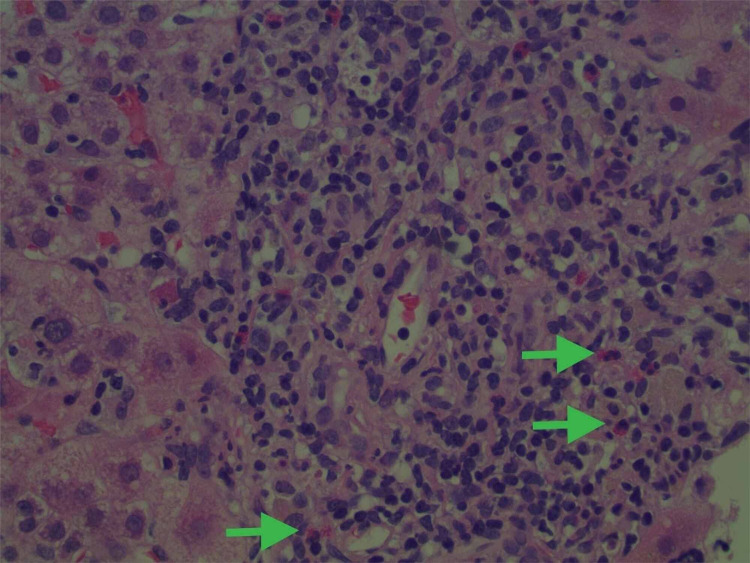
Liver biopsy slide under high power magnification showing chronic portal inflammation Arrows demonstrate eosinophils (pink with granular appearance).

Throughout her admission, multiple tests were conducted to determine the etiology of her acute liver injury. Testing for hemochromatosis gene variants, ceruloplasmin, alpha-1 anti-trypsin, anti-smooth muscle antibody, mitochondrial antibody, anti-nuclear antibodies, and viral hepatitis were all negative or within the normal range as displayed in (Table 1). Ultimately, a liver biopsy was performed and was significant for inflammatory changes with areas of the portal, periportal, and lobular inflammation. There were focal increases of eosinophils within the inflammatory components, further supporting a diagnosis of DILI (Figures 1-2). Additionally, there were areas of cholestasis accentuated in the perivenular regions. The patient’s duloxetine was discontinued with a resulting persistent decline and return of her hepatic enzymes toward baseline, as demonstrated in Table 1 above.

## Discussion

There are typically three patterns of hepatic injury that each has a unique clinical presentation: hepatocellular (hepatitis), cholestatic (cholestasis), or a mixed picture that includes features of both [5]. Our patient presented with a mixed picture due to the disproportionate elevation in the serum aminotransferase when compared to alkaline phosphatase and increased total bilirubin. However, she still demonstrated a significant elevation in alkaline phosphatase with evidence of cholestasis in the perivenular regions on pathology reports. The additional findings of inflammation with the presence of focal eosinophils in biopsy specimens further support DILI over other mechanisms of hepatotoxicity (Figures 1-2).

Unfortunately, specific serum biomarkers or histologic features that can delineate or identify the exact drug inciting acute liver failure do not exist at this time. Therefore, ascribing DILI to a particular drug requires thorough past medical, familial, social, and medication history gathering, ruling out multiple etiologies of the underlying disease, discontinuation of suspected insulting medication, and monitoring the patient’s clinical response-all of which were performed in our patient.

There have been few additional studies published that report a link between the use of duloxetine in patients without liver disease and the development of DILI (Table 2). A review of these cases demonstrates that women are predominantly affected, with a ratio of 8:2, and the mean age of insult is 46.5 years old. Our patient falls into these demographics as a female at the age of 43 years old. However, there is currently no established correlation associated with the sex and age of patients as it relates to the risk of hepatic injury.

**Table 2:** Summary of case studies reporting drug-induced liver injury (DILI)

Study	Age/Sex	Type of Injury	Onset of Symptoms	Risk Factors	Dosage	Intensive care unit (ICU)/Floor	Treatment
Yuan W et al. [6].	58/Female	Acute Hepatic Failure	Two days	-	90 mg/day (7 weeks)	ICU	Protonix, lactulose, IV fluids
Hanje A et al. [7].	56/Female	Fulminant Hepatic Failure	Two weeks	-	30 mg/day (1yr) > 60 mg/day (6 weeks)	Floor	Lactulose, Fresh frozen plasma, Recombinant Factor VIIa with incracranial pressure monitor with persistent deterioration
Vuppalanchi et al. [8].	37/Male	Hepatocellular and cholestatic hepatic injury	Two days	Chronic alcoholism	60 mg/day (6 weeks)	-	-
	28/Female	Acute Hepatic Necrosis	-	-	60 mg/day (1 month)	-	Supportive
	49/Female	Acute Hepatic Necrosis superimposed on non-alcoholic steatohepatitis	-	Non-alcoholic fatty liver disease, Clonazepam	30 mg/day (4 weeks)	-	-
	56/Female	Cholestatic Hepatitis	-	-	30 mg/day (9 days) then dose increased to 60 mg/day (9 weeks)	-	-
	49/Female	Cholestatic hepatitis	-	-	60 mg/day (5 weeks)		-
	58/Female			Chronic hepatitis C, Acetaminophen & Clonazepam	30 mg/day (2 weeks)	ICU	
	52/Female	Acute Hepatitis	-	Methotrexate (3yrs)	60 mg/daily	-	-
Park et al. [9].	22/Male	Cholestatic Jaundice		-	30 mg/daily, 60 mg/daily (3 month)	-	-

Additionally, further review of the data suggests that DILI in duloxetine users occurs in an idiosyncratic manner. Thus, the predictability of potential injury is challenging to assess and concrete recommendations for dose management are challenging to make. The average amount of time before patients reported to a physician with clinical symptoms in DILI cases was approximately six weeks (based on review of prior case studies referenced in Table 2). It may be advisable for physicians interested in starting their patients on this medication or making dose adjustments to perform baseline LFTs at initiation and repeat labs at the four-to-six-week point to monitor medication tolerance. Intermittent monitoring alongside routine labs may also be advisable thereafter, as our patient had been on a long term (five year) course of duloxetine prior to the development of DILI. 

## Conclusions

DILI in patients that take duloxetine without a history of hepatic disease is a rare entity. We report a case of a 43-year-old, otherwise healthy, female with duloxetine-induced liver injury. She presented with an acute liver injury that improved after the discontinuation of duloxetine. Physicians prescribing this medication should be aware of the potential for liver injury, and may consider monitoring LFTs at baseline, at the four-to-six-week period after initiation or adjustment of duloxetine dosage, and intermittently on routine labs thereafter.

## References

[1] Hussaini SH, Farrington EA (2014). Idiosyncratic drug-induced liver injury: an update on the 2007 overview. Expert Opin Drug Saf.

[2] Ostapowicz G, Fontana RJ, Schiodt FV (2002). Results of a prospective study of acute liver failure at 17 tertiary care centers in the United States. Ann Intern Med.

[3] Larson AM, Polson J, Fontana RJ (2005). Acetaminophen-induced acute liver failure: results of a United States multicenter, prospective study. Hepatology.

[4] Chalasani N, Fontana RJ, Bonkovsky HL (2008). Causes, clinical features, and outcomes from a prospective study of drug-induced liver injury in the United States. Gastroenterology.

[5] Batt AM, Ferrari L (1995). Manifestations of chemically induced liver damage. Clin Chem.

[6] Yuan W, Williams B (2012). Acute hepatic failure involving duloxetine hydrochloride. J Neuropsychiatry Clin Neurosci.

[7] Hanje AJ, Pell LJ, Votolato NA, Frankel WL, Kirkpatrick RB (2006). Case report: fulminant hepatic failure involving duloxetine hydrochloride. Clin Gastroenterol Hepatol.

[8] Vuppalanchi R, Hayashi PH, Chalasani N (2010). Duloxetine hepatotoxicity: a case-series from the drug-induced liver injury network. Aliment Pharmacol Ther.

[9] Park YM, Lee BH, Lee HJ, Kang SG (2010). Cholestatic jaundice induced by duloxetine in a patient with major depressive disorder. Psychiatry Investig.

